# Challenges for comparative politics. Virtual conference of the DVPW-Section “Comparative Politics”, October 4th, 2021

**DOI:** 10.1007/s12286-021-00500-w

**Published:** 2021-12-14

**Authors:** Muriel Cathérine Pluschke, Maria Keller, Lea Buchholz

**Affiliations:** 1 Fulda University of Applied Sciences, Leipziger Straße 123, 36037 Fulda, Germany; 2 TUM School of Social Sciences and Technology, Technische Universität München, Richard-Wagner-Straße 1, 80333 München, Germany

The 2021 conference of the research section “Comparative Politics” of the German Political Science Association was entitled “Challenges for Comparative Politics”. It took place digitally on the 4th of October, 2021 and was organized by *Claudia Wiesner* (Fulda University of Applied Sciences), *Norma Osterberg-Kaufmann* (Humboldt University Berlin) and *Stefan Wurster* (Technical University of Munich). In eight panels, the international participants presented their own research, received feedback on their projects and took part in lively discussions. They gathered input on how to overcome the challenges which come up in various topics of comparative politics such as formulating research questions after defining the focus of interest, finding the correct method of comparison, or developing the appropriate research design. Questions which were discussed ranged from the research process to methods in general to specific case studies and analyses. The daylong conference ended with a round table on the topic “Glossing over or zooming in? Beyond Western Monism in Political Science”.

The first panel on the topic of *Doing qualitative research* started with a presentation by *Claudia Wiesner* (Fulda University of Applied Sciences) who was also, along with *Kristin Annabel Eggeling* (University of Copenhagen), one of the panel’s chairs. Under the title of “Doing qualitative research: Reflecting principles and principled challenges” she covered the topic of conducting qualitative research in general while also discussing caveats and problems. Wiesner especially highlighted the importance of reflection, which includes being clear about the position one is taking when doing research and keeping a certain distance to the research topic. She also emphasized the veto power of sources and the necessity of being open to the research findings. To complete her presentation, Wiesner spoke about some exemplary steps in the research process which include the formulation of a research question, the selection of methods, cases and material, the coding and the what/how/why of the findings.

Parliamentary debates were the focus of the second panelist’s research. In his paper “The Politics of Digital Reading. How to use parliamentary debates as sources of political analysis?”, *Kari Palonen* (University of Jyväskylä) presented his analysis of plenary debates in the German Bundestag. He called this analysis a “methodological experiment” because the digitized parliamentary debates available publicly online had to be searched with search options that offer ways of targeted, non-linear reading and the search criteria related to parliamentary politics itself. Therefore, his aim was not to achieve any statistical representativeness, but to collect representative anecdotes and find out more about the uses of the “politics-vocabulary”. Thus, Palonen followed three guidelines: 1) looking for cases in which the political aspect of the question under debate was particularly emphasized; 2) applying his politics-typology (politicization, polity, politicking, policy) to classify actual uses of the “politics-vocabulary” and to sketch historical interpretations in conceptual changes; and 3) the construction of a set of *topoi*, described as rhetorical knot-points around which interesting conceptualizations can be expected. He found nine topoi of politics in the German Bundestag which included, for example, politics as debate or parliamentarians as politicians.

The third panelists’ planned paper dealt with a quite different approach, as it was written as a joint reflection through emails between the two authors, *Kristin A. Eggeling* (University of Copenhagen) and *Richard Freeman* (University of Edinburgh). Their aim was to consider how issues of research methodology can be linked to teaching political science. A topic that has been of even greater importance during the last two years due to the pandemic. The authors’ goal was to find out how to capture the students’ initial excitement about studying politics and not to overload them with theories. They started with four characteristics of interpretivism and tried to translate them into political science pedagogy. The results were five themes or sections that structure their paper: 1) *being a teacher, doing teaching* which focuses on the performativity of teaching, stressing that a teacher should take over a facilitating role instead of an active one; 2) *culture, economy, authority* which deals with the demand to make positionalities transparent; 3) *knowing and learning* which entails helping the students to make their prior knowledge explicit and provide them with an “infra-language”; 4) *teaching interpretively* including the focus on real cases, applying theories instead of starting to discuss topics on an abstract, theoretical level; and 5) *where is politics?* which refers to the idea that politics takes place in the here and now and the need to teach students how they are implicated and how they can initiate change themselves. Eggeling ended her presentation with open questions for further research and reflections such as: “Does any of this resonate with your teaching?”.

The last speaker on the first panel of “Doing qualitative research” was *Baptiste Dufournet* (Unil-GREC). Under the title “Constructing one’s political intentionality as a gay activist: how do mind and institutions shape political actions”, he elaborated on some of his first results and ideas of an analysis based on the research question “How do mind and worldviews enable gay activists to construct an intentionality to act, and why are some worldviews ‘more shared’ than others?”. Using an interpretive analysis to identify meaning, he looked at semi-direct interviews with gay activists and non-activists. Dufournet conducted a critically inspired analysis on the emergence of a dominant discourse and reflected on the social and political effects of what is discussed and what is not. Some of his first findings include that there is a heterogeneous toolkit that can be used to construct worldviews on specific dimensions in contrast to some of the prevailing homogenous worldviews. Dufournet concluded that there are nodes of meaning in the process of shaping intentionality and action, and that institutions can shape these, which leads to some repertoires being more shared than others.

Panel Two was chaired by *Norma Osterberg-Kaufmann* (Humboldt University Berlin) and focused on *Challenges of Researching Attitudes*. The panel was opened by *Bastian Herre* (Our World in Data; University of Oxford) with his contribution “Identifying Ideologies: A Global Dataset of Political Leaders, 1945–2019”. His research has been motivated by the question of whether the economic ideology of political leaders matters for politics. To go beyond the research of OECD countries, he conducted an annual study in 178 countries from 1945–2019 to determine the ideology of political leaders around the globe. Political leaders were classified as leftist, centrist, rightist or non-ideological (which rarely occurred). This was mainly identified through direct personal statements, direct evaluations by secondary sources as well as affiliations with political parties and the leader’s personal background. The paper presents the dataset’s contents, coding and illustrates its uses. Herre found out that most leaders have identifiable ideologies.

The following presentation “Calling the shots: internal and external legitimation in authoritarian regimes during the Corona pandemic” was held by *Aron Buzogany* (University of Natural Resources and Life Sciences, Vienna (BOKU)), *Rolf Frankenberger* (University Tübingen) and *Patricia Graf* (BSP Business School Berlin). Their research focused on legitimation strategies of autocracies during the COVID-19 pandemic. The research is based on the previous finding that dictators strive to stay in power and literature shows that there are different strategies to do so. Thus, the research team looked at narratives of legitimacy and/or ideologies of the regimes in China, Russia and Brazil during the COVID-19 pandemic. The cases were selected as a spectrum from closed (China) to open (Brazil). Different legitimacy strategies were analyzed: input and output as well as internal and external strategies. Thereby, the narratives of legitimization and/or ideologies of the regime needed to be established in the belief system and political culture of the society. Furthermore, they highlight successes (e.g. fast development of the Sputnik vaccine in Russia) and also demonstrate how this leads to conquering the crisis (e.g. low infection rate). So far, the researchers found differences in input- and output-legitimacy between the different autocracies. Ultimately, the results of the research design could be applied to other crises.

The presentation of “Measuring Democratic Legitimacy in Multi-level Governance” by *Andrea Zeller* (University of Koblenz-Landau) closed the second panel. The background and research interest stemmed from a rising authority of International Organizations (IO) and Regional Offices (RO). The aim of the research is to provide means of measuring democratic legitimacy in various IO/RO for member states in relation to a selected policy field. The research is meant to create a better understanding of democratic legitimacy of IO/RO and to contribute to the discussion on democratic legitimacy of Multi-Level Governances.

Panel Three (chaired by *Claudia Wiesner* and *Kristin Annabel Eggeling*) focused on *Texts, Documents and Discourses*. The panel was opened by *Petra Ahrens* (Tampere University) and *Anna Elomäki* (Tampere University) presenting their project “Researching blurry and contentious topics in supranational settings: Gender Mainstreaming (GM) in the European Parliament and its political groups”. Based on feminist institutionalism, social constructivism and micro-politics, the project focuses on GM in the European Parliament (EP). It is expected that GM itself as well as its implications can be detected in any committee and political group. The main source of data are semi-structured expert interviews with MEPs and EP staff. This is complemented by parliamentary ethnography as well as content analysis of various documents related to the EP and political groups. The data is team-coded and then analyzed using Grounded Theory. The first results indicate that it is beneficial to ask “everyone” which provides in depth insights. Additionally, informal rules could be identified as routine processes. However, the researchers faced difficulties as the recruitment turned out to be time-consuming. Furthermore, it is still challenging to grasp gender mainstreaming in processes rather than in policy fields.

The panel was continued by *Anna Malandrino* (University of Bologna) presenting her paper “Combining Social Network Analysis with Quantitative and Qualitative Document Analysis. Exploring the use of discourse analysis in public policy studies in the XXI century”. The goal of her study was to assess the potential of triangulating qualitative and quantitative text analyses with document-based Social Network Analysis (SNA) to predict policy learning and administrative culture change. Some of the first observations were that the intensity and type of relationship between organizations can be captured by SNA. However, repository arrangement issues also occur since there are duplicates and documents are not always (immediately) available. It was identified that document analysis is not originally conceived to be used in research. Thus, it needs to be found out if the research needs to be adapted (e.g. longer time frame) or if additional qualitative data should be added.

*Marina Strezhneva* (The Institute of World Economy and International Relations of the Russian Academy of Sciences (IMEMO)) went on to present the third paper of the panel: “Towards interpretative operationalization of Alexander Bogdanov’s structural-realist vision in political studies”. This research is based on previous findings about how Brexit has been debated in the British parliament. She explained how, at first sight, the discourse looks bizarre which was the impetus for the research project to find out what the debates were actually about. As a result, she found that the debates could be defined as legitimacy games which turned out to be very rational. However, her research was based on Luhmann’s autopoietic systems theory instead of Alexander Bogdanov’s theories. Then, she reflected on Bagdanov’s views to provide a new interpretation. Thus, she used a constructivist epistemology and an individualist methodology as well as the method of abduction.

The last presentation of the third panel was about “Actors, concepts, controversies: Towards a micropolitical approach in EU studies” and was delivered by *Claudia Wiesner *(Fulda University of Applied Sciences). The aim of her research is to propose an alternative perspective on European Integration which concentrates on political concepts and conceptual politics as its driving factor. She argued that European integration is a contingent historical process and has been shaped in political struggles. Thus, light should be shed on ambivalences and pitfalls using a micro-political, speech-act, action-oriented as well as conceptual and historical perspectives, methodologically based on conceptual history by Koselleck, Skinner and Palonen. This approach offers important theory, methodology and tools that allow researchers to re-think concepts like analytical categories and understand conceptual controversies like political controversies. Furthermore, it offers the possibility of studying linkages between institutional, social and conceptual changes. It is also developed to study new political spaces and focus on debates, conflicts and differences in these processes. A micro-political speech-act and action-oriented perspective allows researchers to systematically highlight dynamics and factors of the complex EU integration that have so far been under-researched and cannot be captured by other approaches. Several assumptions of the new approach are that concepts are coined via their usage, that they are used in political controversy and that practices are often textual and legal. As examples, the panelist explained how the European Parliament gained the right to investiture.

The fourth panel was a plenary book panel, chaired by *Anna Fruhstorfer* (University of Potsdam), that dealt with the new publication *Beyond Presidentialism and Parliamentarism: Democratic Design and the Separation of Powers* by *Steffen Ganghof* (University of Potsdam). In the panel, *Jessica Fortin-Rittberger* (University of Salzburg), *Philip Manow* (University of Bremen), *Armin Schäfer* (University of Münster) and *Christian Stecker* (Technical University of Darmstadt) provided their feedback in short presentations after the author himself summed up the book’s content. Despite some points of critique, the panelists overall agreed that this book could become a classic in Comparative Politics. The aim of the book is primarily to make a contribution to institutional, constitutional and democratic theory by introducing semi-parliamentarism as a design option and for the analysis of independent causal effects of the separation of powers. Steffen Ganghof criticized that most theory is formed inductively. Furthermore, he hinted at cognitive-conceptual path dependencies that lead to anomalies that do not apply to situations in political reality. Based on this observation, the author argued for a coherent revision of theoretical concepts on systems of government and types of democracy. His secondary aim was to provide constitutional implications and proposals. For this purpose, he took semi-parliamentarism as a focal point and highlighted the necessary separation of two analytical dimensions whose assessments differ in literature: a) the separation of powers between government and parliament, and b) executive personalism.

A first piece of feedback was provided by *Jessica Fortin-Rittberger*. She found that the book challenges many branches and named four aspects as noteworthy: 1) the challenge to tri-partite vision of forms of government; 2) the challenge to conventional categorization of forms of government; 3) the different prism than the conventional patterns of democracy; and 4) the bold return to preferable forms of government after a long stalemate in the literature. However, she also raised a few points of critique, e.g. the absence of parties, which—according to Fortin-Rittberger—take a back seat in the book and are not really included. *Philip Manow* stressed that democratic typologies have to be rethought and praised the book as a big hit. He raised critique points about the separation of power and legislative control, in which he found conflicting goals between inauguration and parliamentary control as stated in the book. He asked if control was even something that should be ascribed to parliament instead of a parliamentary majority. A question that for him was not really discussed in the book. *Armin Schäfer *meanwhile described the book as mandatory reading in line with Lijphart, Strom and Shugart/Carey. He highlighted that it can help to make sense of cases that do not fit well within other typologies such as Switzerland or Australia. However, he also raised the question of whether Ganghof argued against pure parliamentarism, because—according to Schäfer—parliamentarism does not rely on the separation of powers and cannot balance simple and complex majoritanism. In this regard, presidential and semi-presidential systems may offer a chance for balance. The panel was closed by *Christian Stecker*, a former doctoral student of Steffen Ganghof, who refrained from criticizing the book. Instead, he provided application-oriented input on semi-parlamentarisms in Germany, which he simulated based on election studies. Stecker conducted a simulation based on 106 state election studies from GESIS, which allowed him to discover the real election winner and gain clarity of victory and majority enhancement.

Panel Five (chaired by *Claudia Wiesner* and *Kristin Annabel Eggeling*) covered the topics *Visuals, Practices, Ethnography*. It was opened by *Katja Freistein* (University Duisburg-Essen) and *Frank Gadinger* (University of Duisburg-Essen) who presented their project “Visual Narrative Analysis in the Study of International Politics”. Their object of analysis was images embedded in political narratives. With their narrative analysis, they used a hybrid approach that incorporated visuality, narratives and discourse. Images (as opposed to texts) have the potential of reaching people in a different way since emotions and ideas can be evoked. For the analysis, they used a layering technique (zooming out) to analyze images, then they moved on to the narrative and finally to the larger political context. As an example, they presented an iconic image of Angela Merkel at the G7 in Canada to explain three steps they followed in their analysis: 1. information of the image; 2. context; 3. comparison. In conclusion, they classified their approach as a tool to analyze political narratives even though challenges remain since the researchers started the analysis with their own views and images in mind.

The second topic of the panel “Picturing Politics: Techniques, Challenges and Opportunities for semi-automated online imagery collection in discoursive research” was presented by *Aslak Veierud Busch* (Vrije Universiteit Brussels). He analyzed images as visual representation and narratives using the Arctic as an example. He explained the function of images as well as the fact that images interact with the text around them and also with each other. For his research, he used an exploratory approach by adapting an approach used in tourism studies to analyze how the Arctic is displayed. Here, text and images are separated to see the contours of visual discourses. Therefore, he used Bing to get a (semi-) automated collection of images which he got from side-specific search and then downloaded them in bulk. He concluded that this tool provides a way to tease out a visual discourse in a transformed media landscape. However, this approach has some issues that need to be considered. For example, he argued that methodological debates around digital methods should be grounded in practical experience.

The third presenter of panel five, *Sabine Volk *(Jagiellonian University, Kraków) presented her research on “Being in PEGIDA: An Ethnographic Approach to Studying the Political Culture of Far-right Populist Protest”. Her research is a case study on PEGIDA, which analyzed the question: “How did PEGIDA manage to survive over seven years, even during and beyond the COVID-19 pandemic, lockdown, and despite scarce resources?”. She did “conventional” ethnography by doing participant observation of PEGIDA in 2019 and 2020. This was complemented by a “virtual” ethnography of virtual protest events in real time and digital observation of street events in 2020/2021. In her first findings, she classified PEGIDA as a predominantly anti-left movement, rather than a mainly anti-Islam movement. She also defined PEGIDA as events rather than a pre-defined community as well as a “protest ritual” and a “delayed rebellion”.

The last panelist of panel five, *Katja Mäkinen* (University of Jyväskylä) presented her project “Ethnographic Approaches to EU’s Participatory Governance”. The research was based on the EU’s efforts to promote participation of EU-citizens in EU-programs and the attempt to strengthen its legitimacy through participatory practices. The research focused on the European Heritage Label as one aspect of participatory practice. As a concrete example, the Museum Alcide de Gasperi in Pieve Tesino, Italy, which received the European Heritage Label in 2013, was chosen. In the museum and in the participatory activities organized by it, ethnographic research was carried out. The aim was to find out what meanings the participants give to participation and what roles they give to citizens. This was analyzed by participant observations as well as qualitative semi-structured interviews. From her research, Mäkinen concluded that cultural heritage can provide polyspatial experiences and foster participation through them.

The sixth panel of the working group “Demokratieforschung” on *Challenges in Research on Democracy* started with a presentation by *Toralf Stark* (University Duisburg-Essen), who was also the chair of the panel, *Christoph Mohamad-Klotzbach* (Julius-Maximilians-University Würzburg) and *Norma Osterberg-Kaufmann* (Humboldt University Berlin) on the topic of “Democracy as an ‘essentially contested concept’”. Based on seven criteria of essentially contested concepts, the authors developed three perspectives to look at democracy: 1) a theoretical one which includes the fact that there were major changes in the meaning of and engagement with democracy in the past and there will continue to be changes in the future; 2) a governmental perspective, which shows that democracy is not only contested in the academic debate, but also as a regime type in the political arena; 3) an international perspective which includes the transition paradigm, post-democratization, authoritarian backsliding, as well as good governance and the competition for the best system of government. To start a global search for a core of democracy, the three authors proposed two steps: 1) building “mountains of data” on a global scale to get the whole picture (by focusing on text and people using e.g. surveys, focus groups, …); 2) to examine the “data mountain” with different approaches to find the shared principle of the conceptions of democracy which may exist either as one single principle or as a group or pattern of various principles. The authors highlighted the need for an intra- and interdisciplinary scholarly community, which uses a broad range of methodological tools to create a globally accepted concept of democracy.

*Rolf Frankenberger* (University of Tübingen) proceeded with his presentation on the topic of “What can qualitative research contribute to comparative democracy research?”. He described democracy as not only a scientifically but also a politically contested concept. Frankenberger highlighted the plurality of concepts and understandings that bring about problems of comparing, fine-tuning and developing research instruments. The researcher argued in favor of a more qualitative assessment of democracy-based definitions and perceptions by lay people, politicians and experts as well as theoretical constructs including indices to measure democracy. In addition, Frankenberger mentioned some perspectives for qualitative research, which included the gathering of data from different sources in different formats, the analysis and aggregation of data by constructing categories and by constant comparing, and finally the development of typologies and causal models by linking different concepts. In regards to the last point, he highlighted that vocal expression and actions should be included and that researchers have to stick to the data very carefully while avoiding imposing their own ideas and concepts of democracy, or that they should at least clearly outline them if they wish to include them. Frankenberger also showed some insights from his analysis of speeches by politicians in which “people” emerged as a core feature of democracy. In the following discussion, the need for field research was stressed as only one important point to study.

In his paper “From the Varieties of Democracy to the Defense of Liberal Democracy: V‑Dem and the reconstitution of liberal hegemony under threat”, *Jonas Wolff* (Peace Research Institute Frankfurt (PRIF)) took a detailed look at the Varieties of Democracy (V-Dem) project. His aim was to trace the discursive turn from conceptual contestation to decontestation of democracy in the V‑Dem research and beyond. In his paper, he reconstructed two mechanisms. First, the intra-academic processing of “autocratization”, which includes the perception of threats to democracy and freedom and the worldwide trend of decline in democratic qualities as elements of a discursive turn. This resulted in an unequivocally liberal conception of regime type and regime change by V‑Dem. Second, the policy-oriented knowledge production and dissemination, including a perception of particular threats to liberalism and liberal norms as elements of discursive turn. Here, V‑Dem, using politicization and simplification, ended with a focus on definitive conceptions, numbers and cases and in a turn to an unequivocally liberal understanding of democracy. In addition, Wolff coded 120 V-Dem working papers, which showed that electoral and liberal democracy has become more important while deliberative, egalitarian and participatory declined in relevance over time. To conclude, the panelist argued that conceptual openness is crucial to democracy’s capacity for innovation because the decontestation of democracy as liberal democracy is not only academically but also politically problematic.

The fourth presentation was held by *Anna Fruhstorfer *(University of Potsdam), who talked about “Minority Rights Between Power Sharing and Political Autonomy”. In her research, she answered the research questions: What effect does the process of making a constitution have on the inclusion of minority rights protections? In particular, does more public involvement in the constitution making process lead to more protection of minority rights or do majorities protect their privileged status? Fruhstorfer described the constitutional design process as a multifaceted process of direct democratic involvement, consisting of the three stages of convening, drafting and ratifying. So far, minority rights have been treated as a mixed bag, she argued, although the types of rights are important and the rights therefore should be treated differently. Based on two hypotheses, Fruhstorfer’s findings showed that there is no effort by indigenous self-government, group self-determination and state duty to protect culture and language. However, she was able to show a positive and significant relation between participation in conveying both stage actions and positive actions in regards to transferring wealth or power to oppressed groups, a positive and significant effect of mixed and popular convening on freedom of religion as well as a positive and significant effect of popular convening on equality independent of race. In her conclusion, Fruhstorfer highlighted that public participation has a positive effect on the constitutionalization of several minority rights protections and stressed that public input during the early stages is substantive while at later stages largely cosmetic.

The last presentation in the sixth panel by *Bartek Pytlas* (Ludwig-Maximilians-University Munich) dealt with the topic “Beyond populism: the diversity of thin anti-establishment ideas in turbulent times”. Pytlas stated that in order to better understand the political turbulence in recent decades, researchers need to account for more diverse thin ideas that are used to contest “politics as usual”. He pointed out that these and the (current) political class are not only challenged by populism and technocracy but also by conventional politics. He termed the latter challenges exceptional political vocation, consisting of rhetorical enactment of exceptional political calling, crafts and virtues. Pytlas argued that thin ideas are used as auxiliary mobilization strategies and that one useful strategy is the portraying of one’s supply as both distinct from “politics as usual” and “better” than conventional politics. His research design was based on the goal to comparatively explore the use of thin supply in campaigns of different types of anti-establishment parties (AEP) across time. In his analysis, he looked at 142 social media campaigns during 23 elections in eight EU countries from 2010 to 2019. Pytlas contrasted the rhetorical self-portrayal (what parties say) with more substantive ideological categories (triangulation of literature) and showed that political vocation was the third most salient thin supply behind anti-establishment and people-centrism. Also, political vocation increased in importance for thin supply over time. To conclude, he deemed it crucial to account for diverse ways parties contest “politics as usual” and stressed that anti-establishment contestation can be much more mundane and prosaic.

Panel Seven dealt with the topic of *Comparative Politics and Area Specialization: Making the tensions productive* and was chaired by *Saskia Schäfer* and *Norma Osterberg-Kaufmann* (both from Humboldt University Berlin). It started with a presentation about “Area specialization and universalism in comparative politics. Can the tension be productive?” by *Melis G. Laebens* (Nuffield College, University of Oxford). She explained that contemporary political science has some universalist epistemic foundations. These include universalist theoretical frames, in which the general models guiding human behavior or organizational and societal dynamics are the same, but the parameters are different. She stressed that concepts are often universal but in practice research operates in geographically defined communities of knowledge. Inside these communities there is a common-sensical, shared, implicit or explicit understanding of “how things work”, which can lead to some ideas, theories or phenomena acquiring geographic “belonging”, e.g., Middle East—Islam. Laebens argues that the co-existence of universalist foundational models combined with the notion that the main drivers of politics are different in regions may create undue, exclusive associations between countries or regions and theoretical phenomena. This can have two detrimental consequences: first, a miscategorization due to selectively using the available body of theory and concepts and second, regional scope conditions rather than theoretical ones. After outlining her own experiences with cross-regional research, Laebens concluded that political scientists should be encouraged to think more systematically about the ontological assumptions underlying their theories. She also stressed that cross-regional work may be more rewarding than conventional research design approaches might suggest, and that cross-regional studies can lead to conceptual and theoretical innovation and better specify scope conditions.

The second presentation was titled “Comparative Study of Radicalized Societies and Terrorism: The Cases of India, Israel, and the United States of America” held by *Sangeeta Mahapatra* (German Institute for Global and Area Studies in Hamburg). Mahapatra conducted a comparative study of three self-proclaimed democracies across western and non-western contexts based on the research question: “Are there similarities in norms, causes, and decline of terrorist groups in the three democratic states?” Her findings deduced that the type and time of terrorism in the three countries India, Israel and the US influence normative ethics. She found that identity-based terror (right-wing extremist) was more maximalistic than ideology-based terror (left-wing extremist). Furthermore, she identified common patterns of radicalization and origin, which include similar stages of radicalization and attacks running in cycles and peaking almost two years apart in the three countries. She also found 14 contributing rather than categorical factors of origin e.g. territorial conflict, influence of a charismatic leader, response to state repression and threat to identity, 17 contributing factors for growth of terrorism e.g. local support or funds from crime, and 14 contributing factors for decline of terrorism e.g. internal rivalries, decapitation and reduction in local support or funds. Finally, Mahapatra showed that since 2007–2008, there has been a growth of negative political polarization, religious and racial nationalism, militant populism and co-authoritarianism. According to her, this has resulted in a change from mass-mediated terrorism to hybrid media-enabled radicalization and, in all three countries, to a move from individual/group-based terrorism to mass-scale radicalization.

A focus on Taiwan and Thailand was made in the third presentation by *Janjira Sombatpoonsiri* (Institute of Asian Studies, Chulalongkorn University, Bangkok, German Institute for Global and Area Studies in Hamburg). She presented her paper entitled “Comparing social movements cross-nationally: limits and possibilities” in which she answers the questions: “Why do some young democracies succumb to autocratic return but some can avert it?” and “How do modes of social movement activism shape these two different paths?”. Using a most similar system design with similar conditions but different outcomes, Sombatpoonsiri looked at the two countries in detail. Her findings showed that the absence or presence of institution-building and corrective extra-institutional activism as well as the absence or presence of hybridizer networks (activists in the system) contribute to diversifying the center of power and diluting autocratic influence, which may help to hinder an autocratic return. Furthermore, Sombatpoonsiri found a range of intervening factors such as a reformist faction within former autocratic networks, organized opposition parties, civil society’s ideological alignment with a new democratic order and international threats that propel democratic defense. All of these factors were present in Taiwan, which only showed ominous signs for an autocratic return in 2014. However, in Thailand, ominous signs for an autocratic return were already visible in 2006.

The last presentation was by *Elena Semenova* (Friedrich-Schiller-University Jena) about “Political regimes and longue durée legacies in post-communist countries”. In her research, Semenova looked at the role of legacies as structural longue durée factors in post-communist countries, in which a large variety of political regimes from authoritarian regimes and post-Soviet republics to liberal democracies have emerged since the demise of communism. In the beginning, she criticized that there is no universal theory about the effects of legacy and most scholars only worked on one of the many aspects, e.g. political and institutional, or concentrated on the three most successful cases (Czech Republic, Hungary, Poland). Furthermore, she said that the statistical methods used in the literature were very preliminary and based on simple correlations. To overcome these deficits, Semenova conducted a Prais-Winsten panel regression analysis in 28 post-communist countries for the years between 1990–2020. Additionally, she made use of Bayesian modelling (which uses small samples and highly correlated predictors) to tackle the problems of multi-collinearity. By doing so, she concluded that many factors used in literature do not have any statistical power or were either under- or overestimated. Semenova stressed that the balance of power during the initial period after the demise of communism is important. She also identified the introduction of inclusive political institutions and being situated close to the European border as positive factors, while popularly elected presidents and natural resources were negative ones.

The final panel (chaired by *Iris Reus* (University of Leipzig)) dealt with the topic of *Challenges in Researching Institutions*. It was opened by *Iris Reus* herself and *Oliver Wieczorek* (University of Bamberg) and their project “Mixed Methods in Comparative Federalism Research: Combining Qualitative Content Analysis and Sentiment Analysis to Investigate Differences in the Media Coverage Across the German ‘Länder’ on Covid-19 Measurements”. Their research covers media reports about COVID-19 and raises the question: “When/how much (variation over time) and in which way (sentiments) did the German newspapers report on Covid-19 restrictions?”. Thereby, the focus lies on the restrictions of the Länder using a mixed-methods design. This allowed them to cope with “big data” and at the same time enabled them to identify the content structure of meaning in texts and provide a nuanced picture. In their preliminary findings, the authors identified four periods of political decisions in newspaper reporting: 1) the way into the first lockdown during the first wave of COVID-19; 2) the lockdown; 3) the phase of relaxations; and 4) the time after most of the restrictions had been abolished. Comparing the Länder, different developments and several peeks at different points of time could be identified.

The second paper of the panel “The gender election gap: why women’s underrepresentation persists in France and Germany” was presented by *Agnes Blome* and *Miriam Hartlapp* (both Freie Universität Berlin). This project seeks to analyze the persistent underrepresentation of women in politics in Germany and France. Thus, they are aiming to find out whether women are less likely to be elected than men and how these differences can be explained. The study covered the period between 1980–2017 for Germany and 1993–2017 for France. The data contained individual information on all candidates running for majority vote in all electoral districts. Regression for binary dependent variables were then used to analyze female candidates’ electoral success and female candidates’ nomination in a (un)safe district. In terms of nomination probability, the first findings showed a statistically significant lower probability of women being nominated in safe districts than men. Further findings show that female candidates of the French Conservatives were less likely to be nominated. The incumbency advantage was reduced for female candidates in both countries, being an inheritor, however, increased the chances for female candidates in Germany. Blome and Hartlapp pointed out that there are still some open questions, e.g., why the voter bias is more pronounced in France than in Germany (despite or because of legislative quotas).

The final presentation in panel eight “A New Perspective on Legislative Significance and Legislative Productivity” was given by *Christoph Garwe* (Leibniz University Hannover) who presented the theoretical part of his dissertation. The underlying question of his research was: “How does Legislative Significance affect law production?”. Legislative Significance was first defined as a legislative process highly relevant to all actors involved. The aim of the research is to specify the concept and understand the causal connection between the terms. The research project uses a perspective that utilizes salience to develop an understanding. Legislative Significance thereby is the maximum salience to all involved actors and legislative actors are individual MPs or groups that represent a unified opinion and can oppose or support legislative processes. The assumption is that the legislature has an overall limited capacity of time and resources. The more effortful one process is, the fewer laws there are that can be produced over time. In the process of Legislative Significance, all actors are highly affected by policy changes, which increases conflict since the actors will oppose the changes against their preferences while they support all changes in line with their preferences. Thus, increased effort is necessary to find compromises which leads to the reduction of productivity. Hence, when there are greater policy distances, the process is prolonged and the variance in conflict is dependent on policy distances only. This leads to the conclusion that productivity of the legislative is reduced for significant legislation, conflicts increase for significant legislation and the effect of policy distances on legislative productivity is more directly perceivable.

The conference on the research section “Comparative Politics” ended with a round table on the topic of “Glossing over or zooming in? Beyond Western Monism in Political Science”. It was moderated by *Stefan Wurster *(Technical University of Munich). *Aysuda Kölemen* (Bard College Berlin), *Pinar Bilgin* (Bilkent University) and *Claudia Wiesner* (Fulda University of Applied Sciences) and it discussed whether and to what extent western and in particular German political science takes into account approaches, concepts and methods that are currently debated in the disciplinary discourse around the world—and especially in non-western parts.

The round table started with a first statement by each speaker. Here, Aysuda Kölemen stressed that the topic is very complex and that the current state of the world and existing hierarchies are reflected in academia. Kölemen emphasized that there are publications from all over the world but that most theories and concepts come from Anglo-Saxon institutions while field work and case studies are conducted in other regions, like the Global South. According to her, one reason for this situation might be the dominance of English as the language of science and academia. Pinar Bilgin set a slightly different focus. She said that the current way of integrating pluralism into the study of international politics was not conveniently done and that geo-cultural pluralism may not be the solution. Bilgin argued that with regard to geography, there is a limitation as it is always tied up with the exercise of power and researchers do not always reflect on those limitations. Regarding culture, there are expectations about being different or authentic, which may complicate research. She said that not all of the approaches are equal nor do they carry the same weight. Therefore, multiplicity does not translate into pluralism. Claudia Wiesner argued that German political science that follows the trends set by American universities can have interesting consequences e.g., imported methodological pluralism. Currently, there are very few qualitative studies, and those are mostly interviews. Furthermore, she stressed that the perception of concepts differ around the globe, e.g., the Chinese idea of democracy is completely different to the German one. She also pointed out the importance of relativizing one’s knowledge and being reflective of one’s own western perspective.

Following the first statements, the question was raised whether there is still a western dominance. Aysuda Kölemen argued that the difference in the chosen methods reflects the make-up of the field as, for example, women and non-western researchers tend to use more qualitative studies. However, she found that this would only be a problem if some data were more valuable than other. Pinar Bilgin stressed that western dominance exists not only in terms of theories and methods, but also with regard to the concepts and categories used to make sense of the world. She stressed that researchers should look at their understanding of history and take its limitations into account. Claudia Wiesner added that differences exist even within the European Union, e.g., problems of access to research in Eastern Europe. The round table ended in an open discussion with the audience. Here, the power asymmetries in the world, which are also observable in knowledge production, were highlighted as the central issue. Furthermore, publishing regulations and existing academic norms and structures may hinder progress. Another argument was raised about using a concept only because of its origin. There was a call to all researchers to listen, be open, and to try to include other ideas.

Overall, some key messages can be taken from the conference. Most participants agreed that comparative politics should make use of qualitative forms of research instead of focusing on quantitative research only. The huge variety of methods also demonstrated that it may be beneficial to use new or experimental methods and try new approaches as well as to combine or adapt existing ones to be able to gain further knowledge. Furthermore, political scientists should make use of exchange and debate amongst each other. They should look at comparative political research as a joint project since this conference has shown that a collective approach can lead to new perspectives and promote everyone’s own research. The DVPW conference was also attended by many international researchers, which underpins the ongoing internationalization of the research field.

